# Poly(3,4-ethylenedioxythiophene)-tosylate (PEDOT-Tos) electrodes in thermogalvanic cells†
†Electronic supplementary information (ESI) available: Electrochemical measurements (cyclic voltammetry and impedance spectroscopy), AFM image, and thermal voltage measurement. See DOI: 10.1039/c7ta04891b



**DOI:** 10.1039/c7ta04891b

**Published:** 2017-09-06

**Authors:** Kosala Wijeratne, Mikhail Vagin, Robert Brooke, Xavier Crispin

**Affiliations:** a Department of Science and Technology , Linköping University , Campus Norrköping , S-60174 , Norrköping , Sweden . Email: Xavier.crispin@liu.se

## Abstract

The interest in thermogalvanic cells (TGCs) has grown because it is a candidate technology for harvesting electricity from natural and waste heat. However, the cost of TGCs has a major component due to the use of the platinum electrode. Here, we investigate new alternative electrode material based on conducting polymers, more especially poly(3,4-ethylenedioxythiophene)-tosylate (PEDOT-Tos) together with the ferro/ferricyanide redox electrolyte. The power generated by the PEDOT-Tos based TGCs increases with the conducting polymer thickness/multilayer and reaches values similar to the flat platinum electrode based TGCs. The physics and chemistry behind this exciting result as well as the identification of the limiting phenomena are investigated by various electrochemical techniques. Furthermore, a preliminary study is provided for the stability of the PEDOT-Tos based TGCs.

## Abstract 2

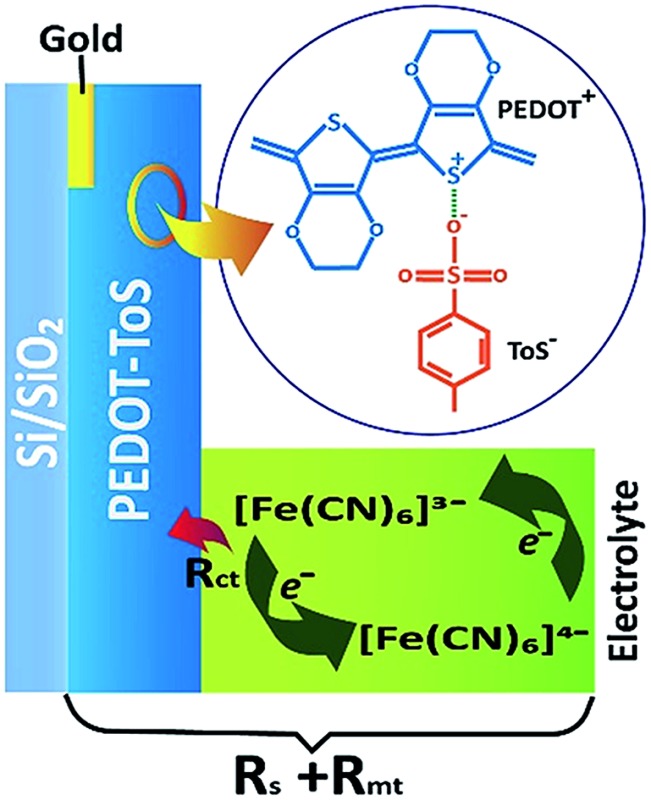
The interest in thermogalvanic cells (TGCs) has grown because it is a candidate technology for harvesting electricity from natural and waste heat. The polymer electrode PEDOT is investigated as potential material to replace Pt electrode in TGCs. The power of the TGC increases with thickness and PEDOT provides an efficient electron transfer to Fe(CN)_6_^3–^.

## Introduction

**Fig. 1:**
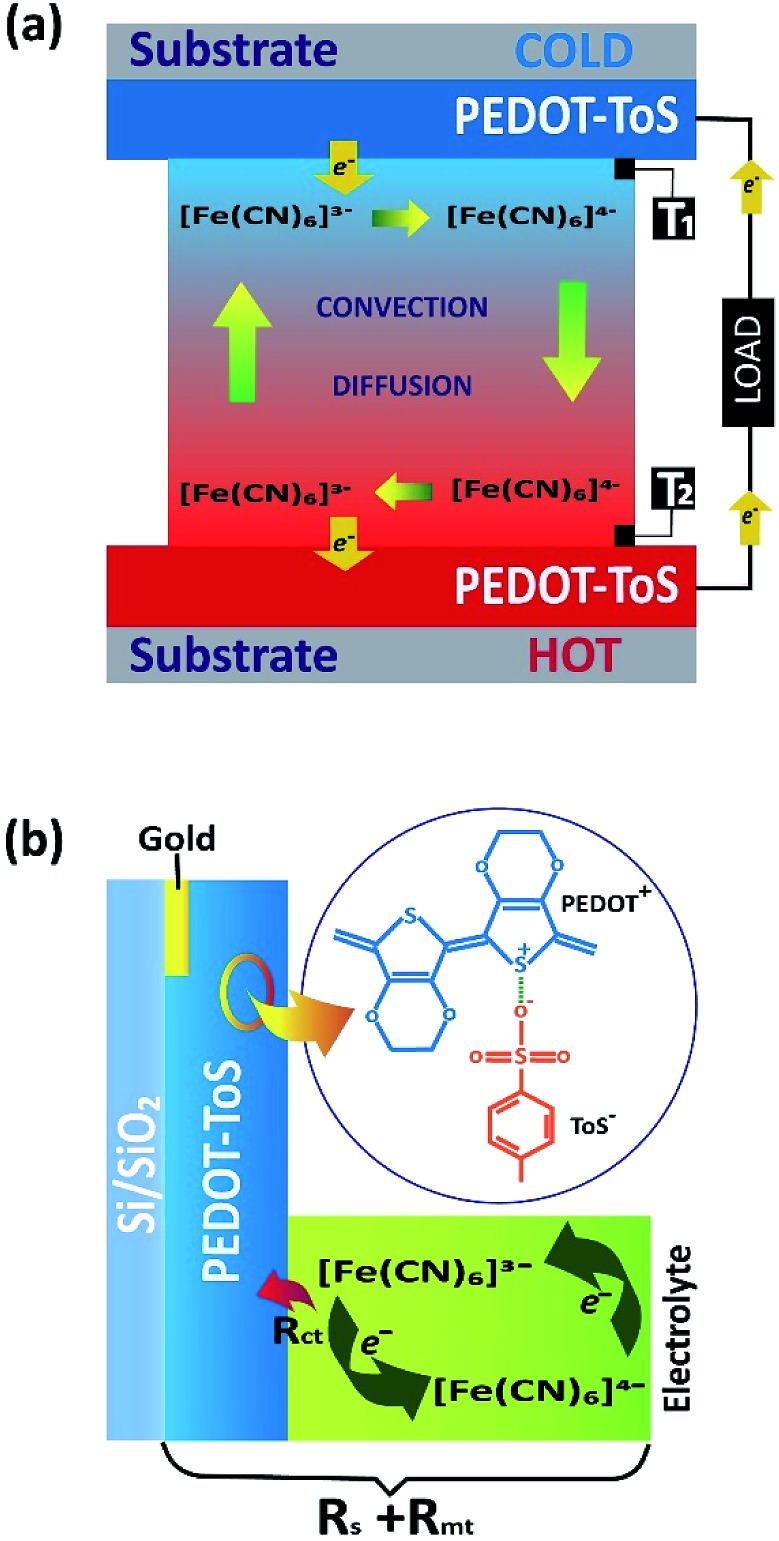
(a) Sketch of the thermogalvanic cell and material/redox electrolyte used (*T*_1_ and *T*_2_ thermo-couples for temperature measurement of the electrode), (b) sketch of ohmic loss contributions of PEDOT-Tos electrode and electrolyte.

Fossil fuels are still the dominant (*ca.* 88%) energy source in our society.^1,2^ A large fraction is used to generate electricity with a heat engine possessing an efficiency approximately 35%. Hence, 65% of the fossil fuel energy is wasted in heat.^3^ Besides that, other large heat sources include solar and geothermal energy, which typically heat materials up to 150 °C.^4^ The growing energy demand and crucial environmental impact of energy systems provide an impetus for effective management and harvesting solutions dealing with waste heat. Beside organic Rankine cycles, a promising way to use waste heat is to directly convert a heat flow into electrical energy by thermoelectric generators (TEGs).^5^ Solid state TEGs are electronic devices without mechanical parts that wear out. Electrical power is generated when charge carriers thermodiffuse in the semiconductor materials with the aid of a thermal field.^6^ While various efficient inorganic materials have been created for high temperature range, such as silicides^7^ and oxides,^8,9^ the best inorganic material for low temperatures (<150 °C) are BiSbTe alloys.^10,11^ Hence, the challenge is to find materials that are composed of elements of high abundancy and high heat-to-electricity conversion efficiency. It is within this arena that organic conductors are making their entrance and are beginning to be scrutinized for TEGs applications.^1,5,12^ However, there is yet another heat-to-electricity conversion device that is under exploration: thermogalvanic cells (TGCs).^13^ TGC is an electrochemical device that consists of two metal electrodes, such as platinum, in contact with the electrolyte solution including both forms of a reversible redox couple (Fig. 1a).^4,14^ The exchange equilibrium between fast oxidation and reduction reaction of the redox couple at the electrode surface establishes a stable electrode potential. In this case, the redox reaction is between [Fe(CN)_6_]^4–^ and [Fe(CN)_6_]^3–^;

1 Fe(CN)_6_^3–^ + e^–^ ↔ Fe(CN)_6_^4–^

A temperature difference (Δ*T*) between the electrodes promotes a difference in the electrode potentials [Δ*E*(*T*)]. Hence, power can be generated by connecting the two electrodes to an external load when they are submitted to a temperature gradient.^15^ The temperature dependence of the electrode potential at steady state defines the Seebeck coefficient *S*_e_ of the redox electrolyte, which is also related to the entropy change during the electron transfer for the redox couple.^16^
2
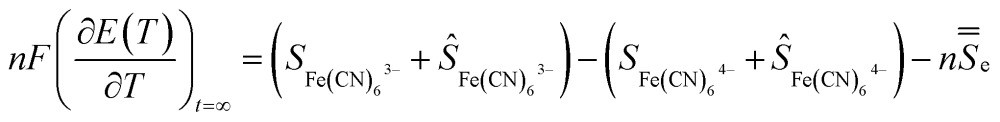
where *n* is number of electrons involved in the redox reaction and *F* is the Faraday constant *S*_Fe(CN)_6__^3–^ and *S*_Fe(CN)_6__^4–^ are the partial molar entropy of species [Fe(CN)_6_]^4–^ and [Fe(CN)_6_]^3–^, *ŝ*_Fe(CN)_6__^3–^ and *ŝ*_Fe(CN)_6__^4–^ are their Eastman entropies of transport, and *S*_e_ is the transported entropy from the electrons in the metallic electrodes. However, the thermodiffusion of the molecular ions is a slow process, which may take several hours to reach steady state. Therefore the practical Seebeck coefficient measured from the open-circuit voltage after 10–20 min represents mostly a difference in partial a molar entropy of the species.^13,17^
3
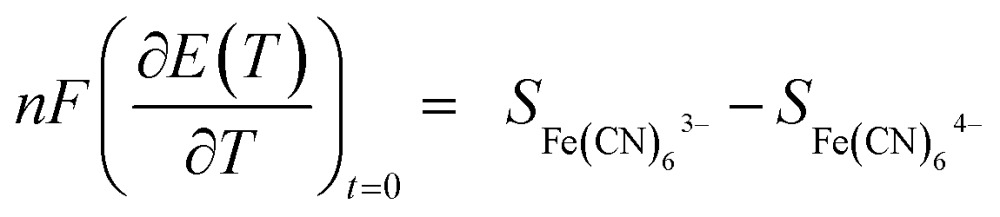



There are several advantages of TGCs compared to TEGs. TGCs provide a higher thermovoltage (∼1 mV K^–1^)^18^ than those achieved in solid state TEGs (∼0.1 mV K^–1^).^19^ The liquid electrolyte employed in TGCs possesses low thermal conductivity (<0.6 W m^–1^ K^–1^)^20^ and can thus maintain a large temperature gradient between the two electrodes in TGCs. The electrolyte is typically composed of elements many orders of magnitude more abundant than bismuth or telluride. The drawback is that the ionic conductivity of the redox species (≈0.3 S cm^–1^)^20^ in a TGC is considerably lower than electrical conductivity in thermoelectric materials (>100 S cm^–1^), which yielded a high ohmic loss. Note that the ohmic loss has several origins in TCGs such as the resistance of the electrolyte and the electrode which collects the series resistance *R*_s_, the mass transfer resistance *R*_mt_ of the redox couple and the charge transfer resistance *R*_ct_ due to the electron transfer between the electrode and the redox couple.^21–23^ Ohmic loss contributions are illustrated in Fig. 1b. In TGCs, the higher the reversibility of the electrochemical reaction (redox current density), the higher the extracted power. Therefore, the main challenge towards the truly scalable thermogalvanic devices is the necessity to have a reversible redox electrode process featured with high redox current. Commonly, electrode materials have been composed of platinum^24–26^ which limits the wide application of the technology simply by material cost. Recently, several reports have shown that the use of porous carbon materials^15,27^ electrodes yield to better performance than platinum electrodes. Carbon based electrodes typically require high temperature synthesis (700–1000 °C);^21,28^ which negates the positive environmental impact of the resultant TGC.

Another class of material, which could provide TGCs with a low environmental impact electrode, is conducting polymers. Their high abundance, low temperature synthesis (<100 °C) and processability through printing technique make conducting polymers an attractive alternative to platinum and porous carbon electrodes. A recent study demonstrated that a film of conducting polymer on stainless steel improved the thermogalvanic device efficiency^16^ without any mechanism clarification. Therefore, the question of whether conductive polymer without supportive metal electrode are suitable electrode materials for thermogalvanics remains unanswered. In this report, we investigate the possibility to use plastic electrodes for thermogalvanic devices incorporating conductive polymers electrodes. We choose the conducting polymer poly(3,4-ethylenedioxythiophene)-tosylate (PEDOT-Tos) which is known to display stability and high electrical conductivity (>1000 S cm^–1^) and can be synthesized by chemical polymerization and vapor phase polymerization^29–32^ PEDOT-Tos electrodes were synthesized by chemical polymerization according to a known recipe and details procedure available in the Experimental section.^1,33^ The performance of thermogalvanic devices were systematically studied using a model 0.4 M K_4_Fe(CN)_6_/K_3_Fe(CN)_6_ redox electrolyte^23,34,35^ with thin film electrodes deposited on insulator and compared to platinum electrodes.

## Experimental section

### Electrode preparation

Platinum electrodes were prepared by thermal evaporation of platinum on silicon wafer (platinum thickness ≈ 120 nm). PEDOT-Tos coated electrodes were prepared by chemical polymerization of EDOT monomer on an electrically insulated silicon wafer (p-type, 1000 nm thermally oxide layer). An oxidant solution composed of 1 ml of iron(iii) *p*-toluenesulfonate (40% solution in *n*-butanol) and 0.04 ml of pyridine was stirrer for 1 h. After that, 0.05 ml of EDOT monomer added into the oxidant solution, mixed well and spin-coated onto the silicon wafer. Then the modified wafer was heated at 100 °C for 10 minutes, washed with excess of ethanol and dried in nitrogen. The thicknesses were increased by the addition of more than one PEDOT-Tos layer. Subsequent polymerizations of EDOT on existing PEDOT-Tos films led to a multilayer structure of PEDOT-Tos which could be used to control the thickness. Thickness of the PEDOT-Tos electrodes were measured by Dektak 3ST profilometer and Atomic Force Microscopy (AFM). The topography images were obtained in tapping mode using a Veeco Dimension 3100 AFM. The morphological images and thickness measurements were analyzed using Gwyddion software.

### Electrochemical characterization

The electrochemical setup consisted of three-electrode configuration with Metrohm Ag/AgCl (3 M KCl) double junction reference electrode and platinum mesh electrode. Cyclic voltammetry was performed in aqueous solution of 10 mM K_3_Fe(CN)_6_/K_4_Fe(CN)_6_ in 1 M KCl as supporting electrolyte room temperature with a computer controlled potentiostat (SP200, BioLogic) using 85% of ohmic drop correction (determined by an impedance measurement at 50 kHz, 20 mV amplitude prior to each voltammetry measurements). Impedance spectroscopy was measured in the frequency range of 500 kHz to 10 mHz with an AC amplitude of 10 mV and impedance measurement was run using 0.4 M K_3_Fe(CN)_6_/K_4_Fe(CN)_6_ with two electrode system including PEDOT-Tos electrode and platinum mesh electrode (1.0 cm electrode separation).

### Thermogalvanic cell apparatus

Thermogalvanic cell measurements were performed using a custom design cylindrical Teflon cell covered with two electrodes. The two circular electrodes have 1 cm in diameter and are distant by 1 cm. Temperatures of both electrodes were controlled by two Peltier elements with feedback from two thermocouples.

## Results and discussion

**Fig. 2:**
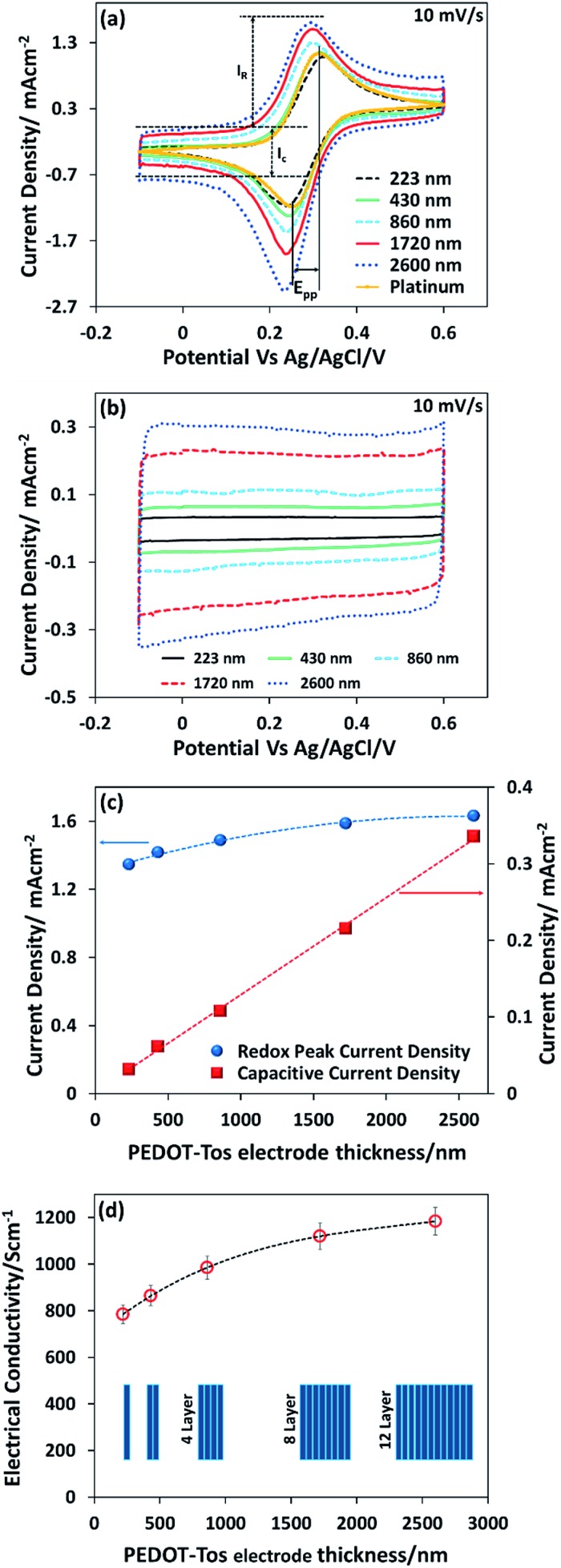
(a) Cyclic voltammetry results of K_3_Fe(CN)_6_/K_4_Fe(CN)_6_ in KCl solution for different thicknesses of PEDOT-Tos electrodes, as well as for platinum electrode. The scan rate is 10 mV s^–1^ (current normalized with the geometric surface area). (b) Cyclic voltammetry of the background electrolyte KCl without redox couple at scan rate of 10 mV s^–1^ (current normalized with the geometric surface area). (c) Plot of the redox peak current density and the capacitive current density as a function of thickness of PEDOT-Tos electrode (current normalized with the geometric surface area). (d) Evolution of the electrical conductivity *versus* thickness.

The conducting polymer is composed of positively charged PEDOT chains and negatively charged tosylate counterions giving the material a polar character. Hence, in contrast to the metal electrode, the solvent molecules and small ions can penetrate into the bulk of the conducting polymer layer forming a porous structure.^36^ However, van der Waals interactions between polymer chains, and electrostatic interactions, have been shown to produce polymer electrodes that are water insoluble.^37^ However it is not known if large molecular ions of the redox couple can penetrate within the bulk of the conducting polymer electrode. To elucidate that, we follow the evolution of voltammetric responses for increasing thickness of PEDOT-Tos, by coating layer by layer from 1 layer up to 12 layers (223 nm to 2600 nm) (Fig. 2a). Note that the voltage range chosen for cyclic voltammetry lies in the stability range of the electrolyte and the PEDOT electrode (ESI Fig. S1†), so the faradaic reaction observed with K_4_Fe(CN)_6_/K_3_Fe(CN)_6_ and PEDOT-Tos electrode is only due to the electron transfer with the iron atoms. The desired thickness is obtained through a multilayer approach (see Experimental). Thin PEDOT-Tos electrodes display a reversible reaction like with Pt that is characterized by two faradic peaks of the same intensity in forward and reverse scans and a small peak to peak (*E*_pp_) voltage (Fig. 2a). Thicker films shows a deviation from this ideal situation with different intensity in forward and reverse scans (Fig. 2a) though a small peak-to-peak voltage. Such observation could be due to an ion exchange between the tosylate ion and iron cyanide, as the redox molecules are present in the film after rinsing the electrode (see in the ESI Fig. S2†). The voltammetry response obtained for conducting polymer electrodes of increasing thickness revealed an enhancement of the both background (*I*_c_) and redox peak (*I*_R_) currents representing, respectively, a capacitive charging and the redox process. The separate measurements have been performed in the absence (Fig. 2b) of redox couple in the background electrolyte in order to elucidate both contributions independently. The capacitive currents *I*_c_ showed a perfect one-to-one linear dependence with the PEDOT-Tos thickness, so increasing by ≈11 times for ≈12 times thicker electrodes (Fig. 2c). Hence, PEDOT-Tos electrode is porous to the supporting electrolyte KCl, and confirming small ions reach all the effective surface area of the electrode upon building the electric double layer. In contrast, the redox peak current slowly rises with the thickness and tends to saturate for thick PEDOT-Tos films (Fig. 2c).

*I*_R_ increases by only ≈1.2 times for ≈12 times the PEDOT-Tos electrode thickness. The difference in thickness dependence between capacitive and redox peak currents suggests that the large redox species [Fe(CN)_6_]^4–^/[Fe(CN)_6_]^3–^ are only slightly penetrating the PEDOT-Tos electrode. This effect however results in a moderate rise in redox peak current for PEDOT-Tos electrode reaching value comparable with the platinum electrode. In order to disregard an effect of increasing surface roughness with the thickness of the PEDOT-Tos electrodes, Atomic Force Microscopy (AFM) was used to characterize the surface morphology (ESI Fig. S3†). No significant difference in morphology (grain size, surface roughness) were observed for the surface of the PEDOT-Tos electrode of various thicknesses. Hence, since the top surface area is constant, the increase in current with thickness suggests either a small surface area increase due to the slight penetration of the redox molecules into the polymer electrode; or that the current is limited by the conductivity of the electrode or other resistive contributions. To investigate more in depth those possible hypothesis, impedance spectroscopy is a tool of choice since it can reveal various resistive contributions by tuning the frequency of the input signal (see next paragraphs). The hypothesis of the impact from the electrode conductivity originates from the observation of an enhanced conductivity of the PEDOT-Tos electrode *versus* thickness by a factor 1.5 (Fig. 2d). Such increase is due to the layer-by-layer polymerization of PEDOT-Tos. In this technique, PEDOT-Tos film was repeatedly exposed to the oxidant and monomer solution, which leads to crosslinking each polymer layer due to the diffusion of oxidant and monomer into the PEDOT-Tos. Both the inter-connection/crosslinking between the polymer layers and the bulk nature might be the reason to increase the conductivity of multi-layer PEDOT.^38–40^

The extrapolation of the redox peak current to the zero thickness yielded the value of redox peak current density of an ideal flat PEDOT-Tos electrode (1.27 mA cm^–2^) of the same order of magnitude obtained for a flat platinum electrode (1.41 mA cm^–2^). One of the possible reasons for the lower current density for PEDOT-Tos *vs.* platinum might originate from the lower electronic density of state at the Fermi level, which is specific of a Fermi glass or semi-metallic character of PEDOT^31^ compared to platinum. The extrapolation of capacitive current to zero thickness provided the value of the electric double layer capacitance on PEDOT-Tos electrode (0.46 mF cm^–2^), which is also the same order of magnitude as on platinum (0.325 mF cm^–2^). The cyclic voltammetry results point out that the multilayer polymerization of PEDOT-Tos electrode is not significantly improve the redox current in an electrochemical device. However, we expect that this effect might be more significant for the TGCs because of the high concentration of the redox molecules.

We compare the performance characteristics of TGCs with PEDOT-Tos and platinum on an insulating substrate. In all the TGCs, the redox electrolyte is 0.4 M K_4_Fe(CN)_6_/K_3_Fe(CN)_6_. This is the benchmark redox electrolyte for thermogalvanic cells. This high concentration of K_4_Fe(CN)_6_/K_3_Fe(CN) is close to its saturation in aqueous medium therefore it is the optimum electrolyte concentration for thermogalvanic cells.^20^ The measured thermovoltage over a temperature range from 23 °C to 53 °C (one substrate heated to 53 °C while other was kept at 23 °C) leads to a Seebeck coefficient value varying between 1.50 mV K^–1^ and 1.43 mV K^–1^ for both PEDOT- and platinum-based devices, which is in good agreement with previously reported values for the same electrolyte.^27,41,42^

**Fig. 3:**
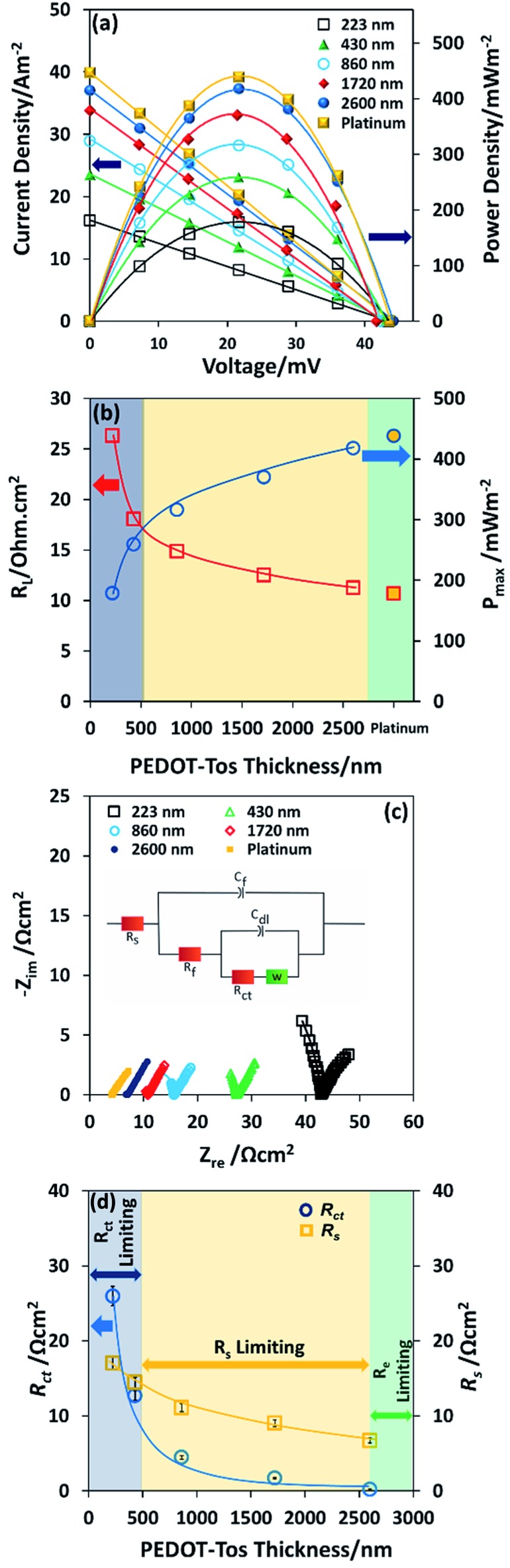
Thermogalvanic cells performance of different thicknesses of PEDOT-Tos and platinum (a) power output and current density as a function of voltage of PEDOT-Tos and platinum, (current normalized with the geometric surface area) (b) comparison of maximum power as a function of PEDOT-Tos thickness and platinum electrode, (c) Nyquist plot for different thicknesses of PEDOT-Tos, the inset shows the equivalent circuit model (d) plot of charge transfer resistance (*R*_ct_) and series resistance (*R*_s_) of PEDOT-Tos electrodes as a function of thickness, the inset shows the series resistance (*R*_s_) dependence with the maximum power density of PEDOT-Tos TGCs.

Note also that the dependence of thermal voltage *versus* temperature difference is identical for different thickness of PEDOT-Tos and similar for the platinum electrode (ESI Fig. S4†). It is seen, that the power output with a single layer of PEDOT (223 nm) is approximately three times lower than that of platinum which is very encouraging (Fig. 3a). An investigation into PEDOT-Tos multilayer and its influence on the TGCs performance was also completed. The power density and current density *vs.* voltage curve are plotted in Fig. 3a. The power increases with the multilayer/thickness from 178 mW m^–2^ (223 nm thickness) up to 417 mW m^–2^ (2.6 μm thickness); which is close to the values obtained with the platinum electrodes (438 mW m^–2^). The dependence of maximum power on PEDOT-Tos thickness (Fig. 3b) was linear up to ≈860 nm and tends to saturate above 1720 nm, yet not fully saturated at 2600 nm. The internal resistance of the device at the maximum power displayed in the Fig. 3b. Charge transfer resistance and ohmic resistance are measured by impedance spectroscopy and extracted according to the equivalent circuit model proposed by Chirea *et al.* ^43^ (see equivalent circuit model display in the inset of Fig. 3c). The Nyquist plot (Fig. 3c) shows that the charge transfer resistance (*R*_ct_) diminishes from 25.94 Ω cm^2^ to 0.22 Ω cm^2^ following the thickness increase. ESI Fig. S5–S8† display the readjusted scale of the Nyquist plot of PEDOT-Tos (860 nm, 1720 nm and 2600 nm) and platinum electrode. Furthermore, the series resistance (*R*_s_) reduces for thick plastic electrodes which includes the resistance of the electrolyte solution (*R*_l_), and the electrode contact resistance at the electrode material and current collector interface. Charge transfer resistance (*R*_ct_), series resistance (*R*_s_), double layer capacitance (*C*_dl_) Warburg Impedance (*Z*_d_), bulk electrode resistance (*R*_f_), and bulk redox capacitance (*C*_f_) are listed in ESI Table S1.† The maximum power increase with the thickness is due to the reduction of charge transfer resistance and series resistance; which are major limiting factor for the power in a thermogalvanic cell. There are two origins for the decrease in charge transfer resistance with thickness: (i) the penetration of the redox couple into the polymer electrode; (ii) the increase in electrical conductivity of the electrode.^41^ Both effect of thicknesses on the resistances *R*_ct_ and *R*_s_ are promoting the power density in the TGC. Fig. 3d illustrates the behavior of the TGCs limiting factors with the increase of PEDOT-Tos thickness and explains the measured internal resistance of the device in Fig. 3b. For thin PEDOT-Tos electrodes, the charge transfer resistance is dominating and limits the power of the TGC. However, for PEDOT-Tos electrodes thicker than a bilayer (>500 nm), TGCs power density is limited due to the series resistance (*R*_s_). Eventually for thick electrodes, the electrolyte resistance become the limiting factor for thermogalvanic performance since electrode resistance decreases with thickness. Note that all the impedance measurement are performed with the same electrolyte concentration (0.4 M K_4_Fe(CN)_6_/K_3_Fe(CN)_6_) therefore the electrolyte resistance (*R*_l_) is identical for all those measurements. Therefore, *R*_s_ is mostly depend on the electrode contact resistance, which decreases with thickness.^44,45^ The large drop in *R*_ct_ (118 folds) with the polymer thickness reveals a potential for further improving the power of the TGC. However, in our device geometry and in cyclic voltammetry set-up, the current seems to be limited by the other resistive components. The high power obtained for thick PEDOT-Tos films originate from the high conductivity and smaller electrode resistance. The extrapolation to the zero thickness gives a maximum power value of 101 mW m^–2^, which is lower but still of the same order of magnitude as that on flat platinum (438 mW m^–2^). This reduction is rationalized to a high electrode resistance and lower electron transfer rate with PEDOT-Tos compared to platinum.

**Fig. 4:**
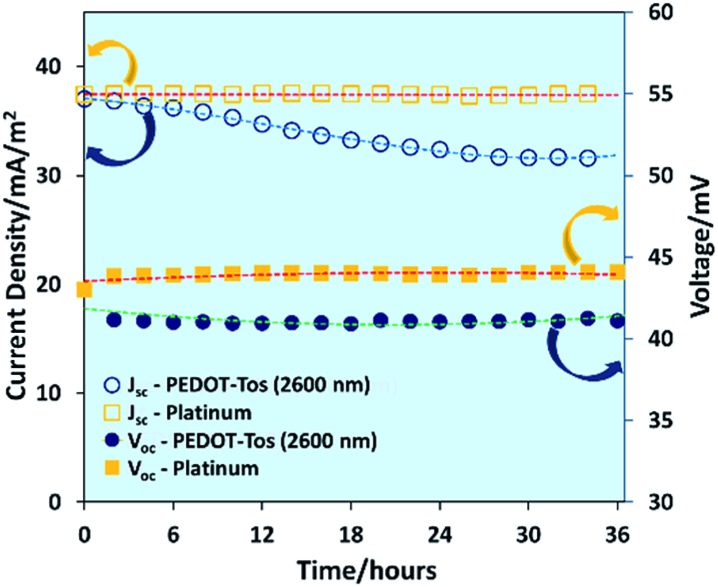
Dependence of open-circuit voltage and short-circuit current density of platinum and PEDOT-Tos (2600 nm) on time with a temperature difference of 30 °C.

The stability of PEDOT-Tos (2600 nm) electrode and platinum electrode TGCs were measured by measuring open circuit voltage and current density for 36 hours. As shown in Fig. 4, the platinum-based TGC displays no change in *V*_oc_ or *J*_sc_ *versus* time. However, for PEDOT-Tos-based TGCs both *V*_oc_ and *J*_sc_ decrease by 5% and 15% after 36 hours. Interestingly, *V*_oc_ of PEDOT-Tos-based TGC drops by 5% from its initial value (43 mV) within the first two hours and then maintain a stable value (41 mV) for entire period. *J*_sc_ of PEDOT-Tos TGC diminishes by 15% of its initial value and after 30 hours it maintains a stable value. Unlike platinum electrodes, PEDOT-Tos electrodes display some degradation over the time but after 30 hours, TGC shows a stable behavior. This might be due to slow ion exchange reaction rather than true chemical degradation, but more research needs to be done to prove the real mechanism behind this observation.

## Conclusions

To conclude, we report for the first time thermogalvanic cells including only conducting polymer films as electrodes. The PEDOT-Tos-based thermogalvanic device performance was optimized by increasing the thickness of the plastic electrode. The efficiency of PEDOT-Tos compared to platinum in the thermogalvanic cell is rationalized by two phenomena. Firstly, the electron transfer occurring between the organic electrode and the redox couple is efficient. Indeed, we found that the current and power density extrapolated at zero thickness for the plastic electrodes are surprisingly high and the same orders of magnitude than platinum. In contrast to metal electrodes, PEDOT-Tos doesn't form any insulating oxide surface layer and thus forms an intimate with the electrolyte. Secondly, the partial penetration of [Fe(CN)_6_]^4–^/[Fe(CN)_6_]^3–^ by diffusion inside the plastic electrodes offer an effective increase of the surface area of the PEDOT-Tos electrodes. The incorporation of PEDOT-Tos electrodes may open new routes for applications of thermogalvanic cells when a flexible substrate is necessary, *e.g.* upon harvesting body heat.

## Conflicts of interest

There is no conflicts to declare.
