# Supplementary material for: Poly(3,4-ethylenedioxythiophene)-tosylate (PEDOT-Tos) electrodes in thermogalvanic cells
Source: J Mater Chem A Mater. 2017 Sep 6;5(37):19619–25. doi: 10.1039/c7ta04891b (PMC5735355; doi:10.1039/c7ta04891b)
Supplement: Supplementary file 1 [file TA-005-C7TA04891B-s001.pdf]

Electronic Supplementary Material (ESI) for Journal of Materials Chemistry A.
This journal is © The Royal Society of Chemistry 2017

Electronic Supporting Information

Poly(3,4-ethylenedioxythiophene)-Tosylate (PEDOT-Tos) electrode in Thermogalvanic Cells

Kosala Wijeratne, Mikhail Vagin, Robert Brooke and Xavier Crispin*

*Email: xavier.crispin@liu.se

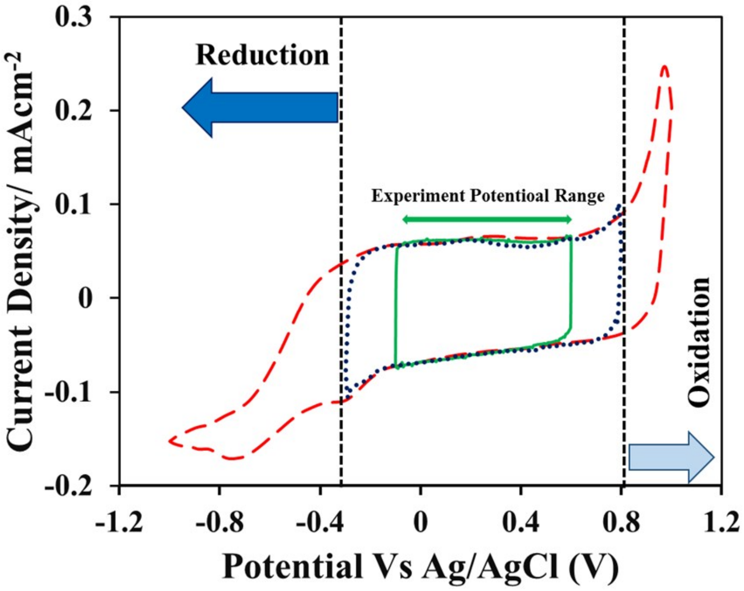

Figure S1 Cyclic voltammetry of the background electrolyte KCl without redox couple at scan rate of 20 mV/s to display the PEDOT-Tos reaction potential.

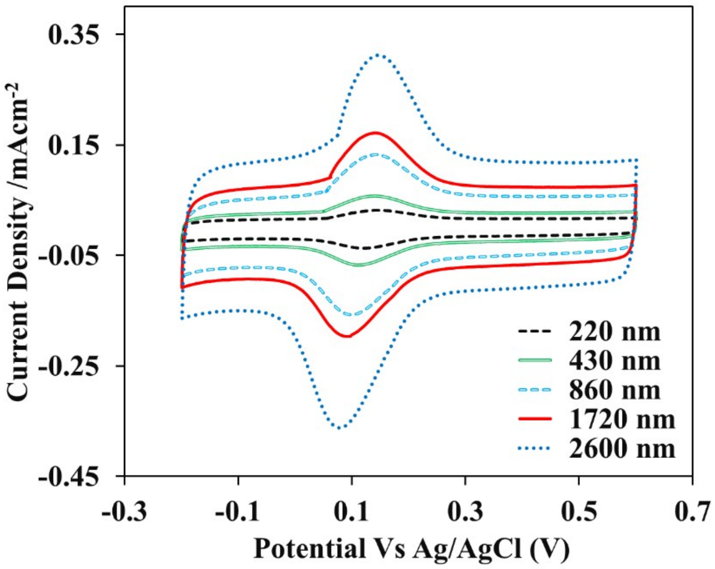

Figure S2 Cyclic voltammetry of the redox couple adsorbed PEDOT-Tos in background electrolyte KCl at scan rate of 10 mV/s.

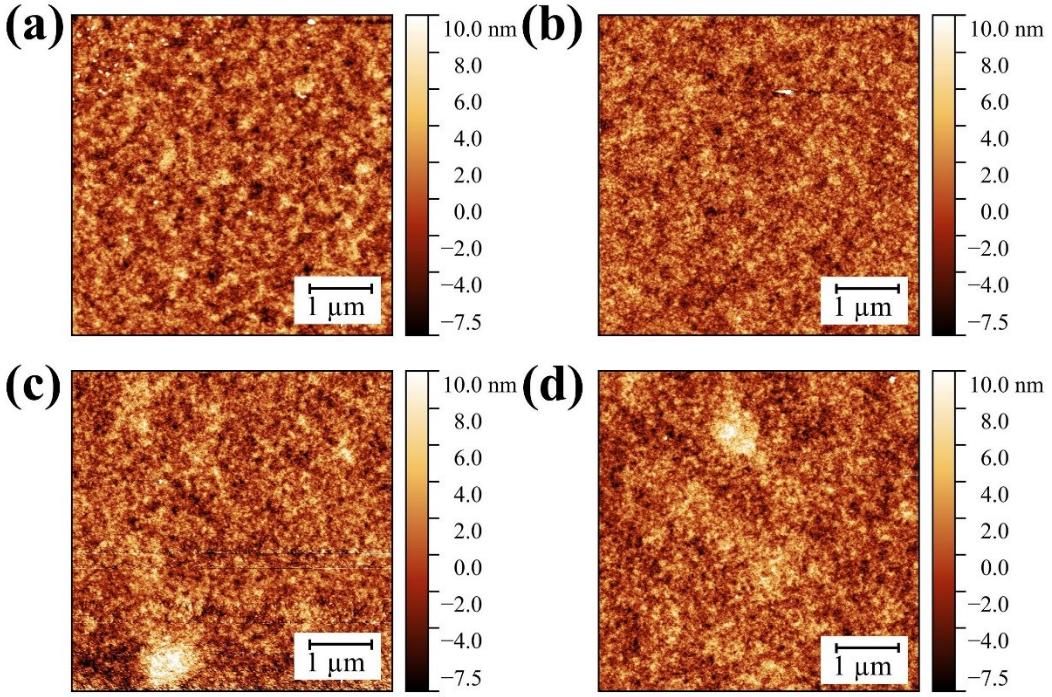

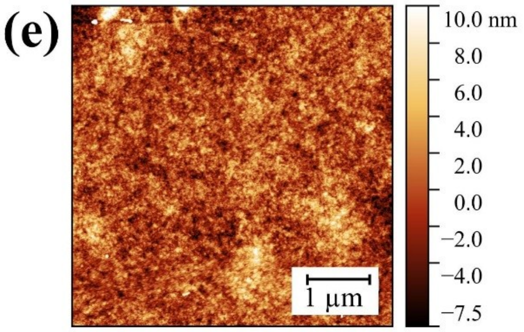

(f)

Layers	Surface Area (um^2)	Roughness (RMS) (nm)
1	25.3	2.59
2	25.4	2.24
4	25.9	2.63
6	25.5	2.48
10	25.3	1.99

Figure S3. Topography images of different thickness of PEDOT-Tos (a) 223 nm, (b) 430 nm, (c) 860 nm, (d) 1720 nm, (e) 2600 nm

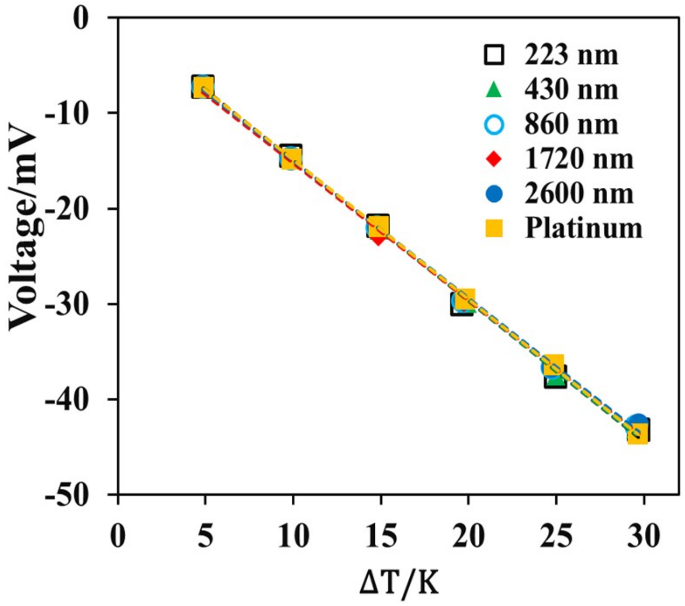

Figure S4. Dependence of thermal voltage on temperature difference for different thickness of PEDOT-Tos electrode.

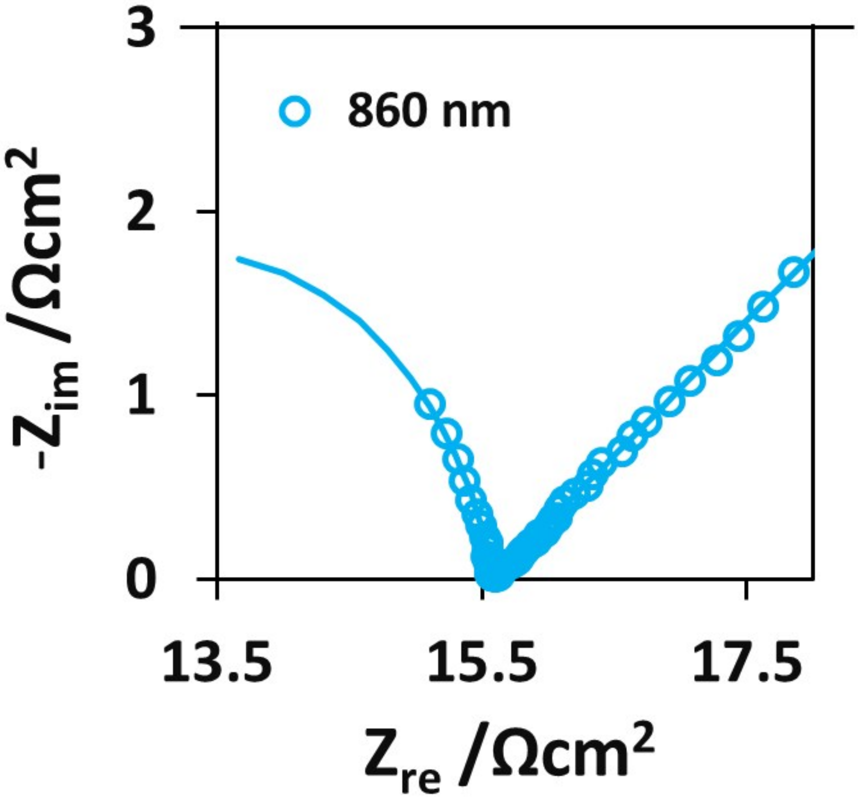

Figure S5. Nyquist plot for 860 nm PEDOT-Tos electrode

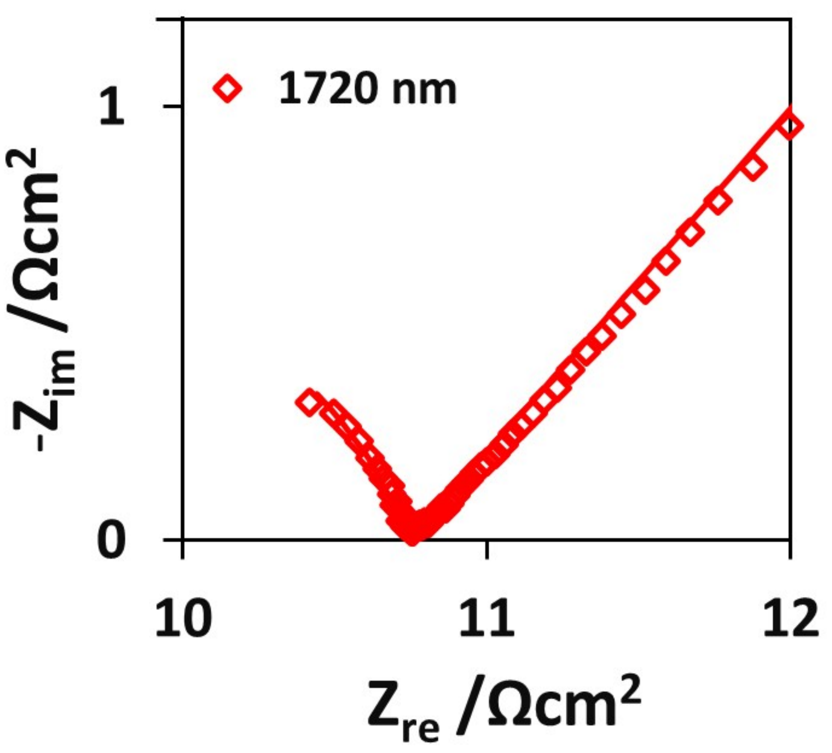

Figure S5. Nyquist plot for 1720 nm PEDOT-Tos electrode

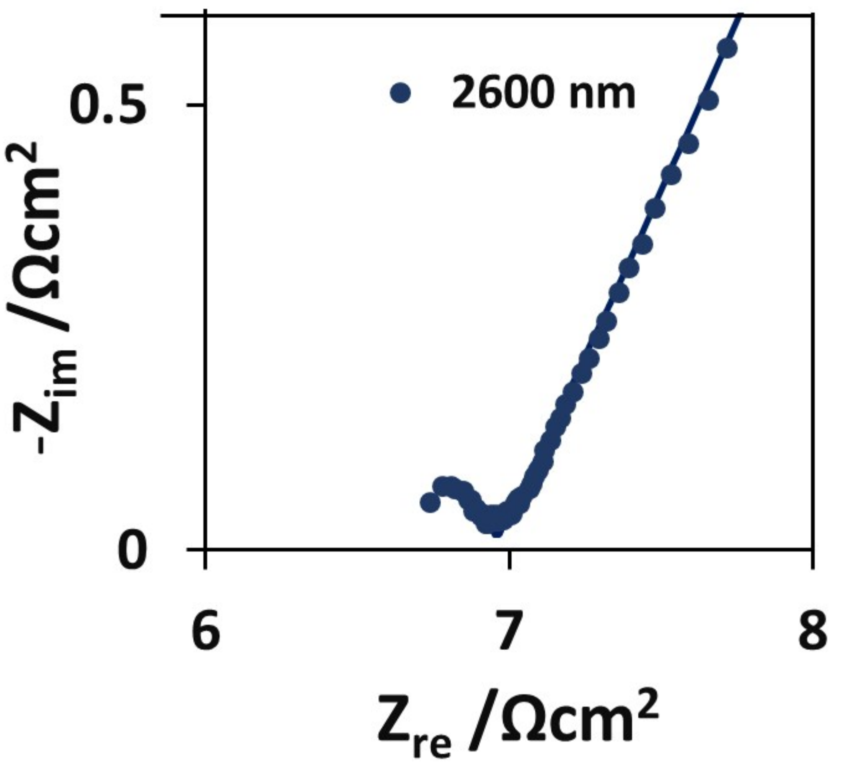

Figure S7. Nyquist plot for 2620 nm PEDOT-Tos electrode

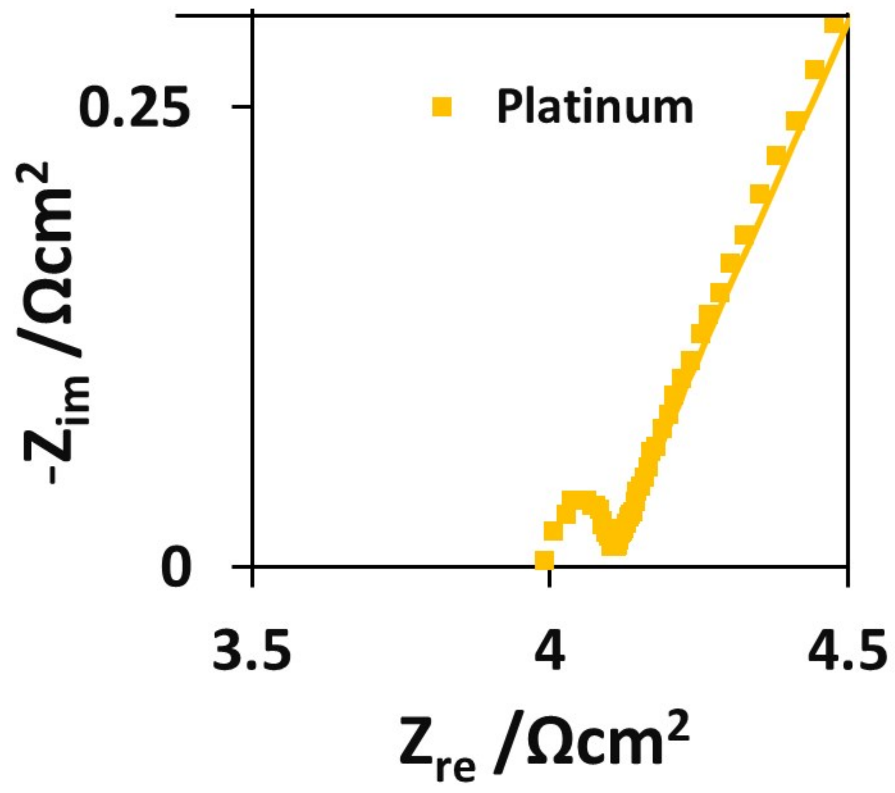

Figure S8. Nyquist plot for Pltinum electrode

Table S1. Impedance Spectrocopy analysis results of different thickness of PEDOT-Tos electrodes

Electrode thickness/nm	R_s/ Ω.cm^2 (+/- 2%)	R_{ct}/ Ω.cm^2 (+/- 2%)	R_f/ Ω.cm^2 (+/- 2%)	C_{dl}/μF/cm^2 (+/- 2%)	C_f/F/cm^2 (+/- 2%)	W/Ωs$^{(-1/2)}$.cm^2 (+/- 2%)
223	17.00	25.94	31.15	0.044	0.544	220.25
430	14.46	12.72	17.05	0.18	0.728	171.48
860	11.10	4.48	11.02	0.58	0.803	64.53
1720	9.02	1.72	5.66	5.89	0.848	13.05
2600	6.73	0.22	3.52	86.3	0.861	6.95
Platinum	4.00	0.086	0.063	23.9	0.827	2.89
